# Angiotensin II Decreases Endothelial Nitric Oxide Synthase Phosphorylation *via* AT_1_R Nox/ROS/PP2A Pathway

**DOI:** 10.3389/fphys.2020.566410

**Published:** 2020-09-30

**Authors:** Jing Ding, Min Yu, Juncai Jiang, Yanbei Luo, Qian Zhang, Shengnan Wang, Fei Yang, Alei Wang, Lingxiao Wang, Mei Zhuang, Shan Wu, Qifang Zhang, Yong Xia, Deqin Lu

**Affiliations:** ^1^Department of Pathophysiology, Guizhou Medical University, Guiyang, China; ^2^Guizhou Provincial Key Laboratory of Pathogenesis and Drug Research on Common Chronic Diseases, Guizhou Medical University, Guiyang, China; ^3^Department of Pathology, The Second Clinical Medical School of Inner Mongolia University for the Nationalities, Yakeshi, China; ^4^Department of Cardiology, The Second Provincial People’s Hospital of Gansu, Lanzhou, China; ^5^Department of Cardiology, Affiliated Hospital of Guizhou Medical University, Guiyang, China; ^6^Department of Neurology, Affiliated Hospital of Guizhou Medical University, Guiyang, China; ^7^Key Laboratory of Medical Molecular Biology, Guizhou Medical University, Guiyang, China; ^8^Davis Heart and Lung Research Institute, The Ohio State University College of Medicine, Columbus, OH, United States

**Keywords:** angiotensin II, angiotensin II type 1 receptor, protein phosphatase 2A, endothelial nitric oxide synthase, NADPH oxidase

## Abstract

Increasing evidences suggest that angiotensin (Ang) II participates in the pathogenesis of endothelial dysfunction (ED) through multiple signaling pathways, including angiotensin type 1 receptor (AT_1_R) mediated NADPH oxidase (Nox)/reactive oxygen species (ROS) signal transduction. However, the detailed mechanism is not completely understood. In this study, we reported that AngII/AT_1_R-mediated activated protein phosphatase 2A (PP2A) downregulated endothelial nitric oxide synthase (eNOS) phosphorylation *via* Nox/ROS pathway. AngII treatment reduced the levels of phosphorylation of eNOS Ser1177 and nitric oxide (NO) content along with phosphorylation of PP2Ac (PP2A catalytic subunit) Tyr307, meanwhile increased the PP2A activity and ROS production in human umbilical vein endothelial cells (HUVECs). These changes could be impeded by AT_1_R antagonist candesartan (CAN). The pretreatment of 10^−8^ M PP2A inhibitor okadaic acid (OA) reversed the levels of eNOS Ser1177 and NO content. Similar effects of AngII on PP2A and eNOS were also observed in the mesenteric arteries of Sprague-Dawley rats subjected to AngII infusion *via* osmotic minipumps for 2 weeks. We found that the PP2A activity was increased, but the levels of PP2Ac Tyr307 and eNOS Ser1177 as well as NO content were decreased in the mesenteric arteries. The pretreatments of antioxidant N-acetylcysteine (NAC) and apocynin (APO) abolished the drop of the levels of PP2Ac Tyr307 and eNOS Ser1177 induced by AngII in HUVECs. The knockdown of p22phox by small interfering RNA (siRNA) gave rise to decrement of ROS production and increment of the levels of PP2Ac Tyr307 and eNOS Ser1177. These results indicated that AngII/AT_1_R pathway activated PP2A by downregulating its catalytic subunit Tyr307 phosphorylation, which relies on the Nox activation and ROS production. In summary, our findings indicate that AngII downregulates PP2A catalytic subunit Tyr307 phosphorylation to activate PP2A *via* AT_1_R-mediated Nox/ROS signaling pathway. The activated PP2A further decreases levels of eNOS Ser1177 phosphorylation and NO content leading to endothelial dysfunction.

## Introduction

Angiotensin (Ang) II is a key component of the renin–angiotensin system and participates in cardiovascular disease (CVD) *via* its specific AngII type 1 receptor (AT_1_R; Tassone et al., 2013; Ding et al., 2016). Although numerous studies have shown that AngII/AT_1_R regulates the physiological and pathological cardiovascular systems, the exact mechanisms involved remain unclear.

Nitric oxide (NO) is the primary endothelium-derived relaxing factor, which is synthesized by the endothelial nitric oxide synthase (eNOS) that plays a pivotal role in regulating endothelium-dependent vessel dilation (Zhao et al., 2015). Altered eNOS/NO function is a common feature of endothelial dysfunction (ED), and the mechanism underlying ED may be related to decreased eNOS activity accompanied by reduced NO production and bioavailability (Lovren and Verma, 2013; Godo and Shimokawa, 2017). Regulation of eNOS activity is complex and involves a variety of mechanisms, such as phosphorylation/dephosphorylation, which is important for post-translational regulation of eNOS. Phosphorylation of eNOS at serine 1177 site (Ser1177), which activates eNOS, was shown to determine eNOS activity regulation in response to various physiological and pathological stimuli (Searles, 2006; Fleming, 2009).

Previous studies have confirmed that activation of AngII/AT_1_R downregulates phosphorylation of eNOS Ser1177 and leads to ED in human umbilical vein endothelial cells (HUVECs; Tassone et al., 2013). Protein phosphatase 2A (PP2A) is the major enzyme that dephosphorylates eNOS Ser1177, and increased PP2A protein expression or enzyme activity resulted in dephosphorylation of eNOS at Ser1177 (Mount et al., 2007; Zhang et al., 2012). Studies have shown that AngII upregulates PP2A activity (Everett et al., 2001; Liu et al., 2015; Li et al., 2016). However, it is not completely clear how the AngII/AT_1_R pathway activates PP2A.

The increased reactive oxygen species (ROS) production (oxidative stress) has been demonstrated to contribute in ED. It is well-established that AngII/AT_1_R can activate NADPH oxidase (Nox) and promote the production of ROS (Liu et al., 2016). The superoxide derived from Nox is a significant stimulator of PP2A (Han et al., 2010). However, it is still unclear whether the AngII/AT_1_R pathway can activate PP2A *via* Nox. Therefore, the aim of the present study was to determine the role of Nox/ROS in AngII/AT_1_R-induced PP2A activation to explore the mechanism of endothelial dysfunction induced by AngII.

## Materials and Methods

### Materials

Fetal bovine serum (FBS; Biological Industries, CT, United States), 0.25% trypsin, high-glucose Dulbecco’s modified Eagle’s medium (DMEM), and cyan-streptomycin were all purchased from HyClone (UT, United States). *N*-acetylcysteine (NAC), apocynin (APO), and AngII were purchased from Sigma-Aldrich (St. Louis, MO, United States). Candesartan (CAN) was purchased from Selleck (Houston, TX, United States). Antibodies against eNOS and eNOS Ser1177 (Millipore, Billerica, MA, United States), PP2Ac Tyr307, p22phox, and PP2A Cα were purchased from Santa Cruz Biotechnology. NO assay kit for HUVECs (DAF-FM DA) and for tissues (Griess reaction), and okadaic acid (OA) was purchased from Beyotime Biotechnology (Shanghai, China). Primary antibody against β-tubulin and secondary antibodies were purchased from PMK Biotechnology (Wuhan, China). ROS assay kit was purchased from Nanjing Jiancheng Bioengineering Institute. siRNA targeting p22phox was purchased from Santa Cruz Biotechnology.

### Cell Cultures

Human umbilical vein endothelial cells were isolated from the umbilical cords of newborns born at the Affiliated Hospital of Guizhou Medical University. Written informed consent was obtained from all participants prior to being included in the study. The study was approved by the Ethics Committees of Guizhou Medical University. HUVECs were isolated as described previously (Hastie et al., 2016; Luo et al., 2019). Isolated HUVECs were seeded and cultured in 6 cm cell culture dishes with using culture medium containing 20% FBS. Expression of factor VIII-related antigen and CD34 was measured using immunohistochemical assay for cell characterization. HUVECs were cultured in 10% FBS in high-glucose DMEM at 37°C in a 5% CO_2_ incubator. When cells reached 70–80% confluence, the medium was replaced with fresh medium for subsequent treatments. HUVECs were used for experiments at passages 4–8.

### Cell Experiments

In order to determine the concentration and duration of AngII, HUVECs were incubated with AngII for 12 h at concentrations of 10^−5^, 10^−6^, 10^−7^, and 10^−8^ M, and in the presence of AngII at the concentration of 10^−7^ M for 6, 12, 24, or 36 h.

Candesartan (CAN) is an angiotensin receptor antagonist that can specifically block the binding of AngII to its specific type 1 receptor. *N*-acetylcysteine (NAC) is a thiol compound, as a donor of cysteine leading to replenishment of glutathione and thus can be used as a reactive oxygen scavenging agent (Aldini et al., 2018). Apocynin (APO) predominantly acts as an antioxidant in endothelial cells and vascular smooth muscle cells (Heumüller et al., 2008). Okadaic acid is a lipophilic natural compound originally isolated from the marine black sponges *Halichondria okadaii* and *Halichondria melanodocia* and is the most widely used inhibitor of PP2A and PP1, with IC50 values of 2 × 10^−10^ and 2 × 10^−8^ M (Dounay and Forsyth, 2002). According to the reports, in the present study, CAN pretreated HUVECs 3 h before the AngII at concentrations of 10^−6^ M to block the binding of AngII to AT_1_R (Wang et al., 2015); NAC and APO pretreated, respectively, 1 h before the AngII at concentrations of 10^−3^ M (Kadowaki et al., 2015) and 2 × 10^−5^ M (Qin et al., 2017) to reduce ROS content; and OA was added to HUVECs 1 h before the AngII at concentration of 10^−8^ M to inhibit PP2A enzyme activity (Chao et al., 2014).

### Animal Studies

All animal procedures were conducted in accordance with the guidelines issued by the Guizhou Medical University Animal Care and Use Committee. Forty-eight male Sprague–Dawley rats weighing 160–200 g were provided by the Experimental Animal Center of Guizhou Medical University [animal certificate number: SCXK (QIAN) 2012-0001] and subjected to adaptive feeding for 1 week. The rats were randomly divided into four groups: Control, AngII, AngII + CAN, and CAN, *n* = 12 in each group. All rats had an osmotic minipump (Alzet model 2002, Alza, Vacaville, CA, United States) implanted subcutaneously in the back of the neck as follows. After intraperitoneal injection of 3% sodium pentobarbital (1 ml/kg body weight), rats were fixed in the prone position on an operating table. A surgical scalpel was then used to make a 1-cm incision behind the ear, over the shoulder that was perpendicular to the tail. A vascular clamp was used to make a subcutaneous pocket for the osmotic minipump. The minipump was inserted gently, the skin incision was sutured, and then mopirocin ointment (Baiduobang) was applied to the incisions for 3 days to prevent infection. Rats in the AngII and the AngII + CAN groups were received AngII *via* the minipump continuously for 2 weeks, whereas rats in the Control and CAN groups were infused with normal saline. The average infusion rate was 500 ng/kg/min. CAN was administered after the minipump implantation by gavage at a dose of 10 mg/kg/day during the AngII infusion. Systolic blood pressure was measured by tail cuff plethysmography with the aid of a computerized system (BP600A, Techman Soft, Chengdu, China) on days 3, 7, and 14 after implantion of the pump.

### Western Blot Analysis

Western blot analysis was performed to measure protein expression. Briefly, radio-immunoprecipitation assay (RIPA) lysis buffer was used to collect total protein from HUVECs and mesenteric arteries. One 6-cm-dish of cells were lysed with 150 μl of RIPA lysis buffer, and 0.1 grams of mesenteric arterial tissue were lysed with 100 μl of RIPA lysis buffer, lysed on ice for 45 min, and then centrifuged at 12,000 *g* at 4°C for 25 min. The supernatants were collected, and protein concentrations were determined. Next, proteins were separated by 10% sodium dodecyl sulfate polyacrylamide gel electrophoresis and then transferred onto transfer membranes (Millipore). After blocking with 5% nonfat milk for 60 min at room temperature, the membranes were incubated with targeted primary antibodies overnight at 4°C. After washing in tris-buffered saline with Tween-20 (TBST), the membranes were incubated with horseradish peroxidase-conjugated secondary antibodies for 1 h at room temperature. After washing the membranes three times in TBST, protein bands were detected using enhanced chemiluminescence reagents (Bio-Rad). Densitometric analysis was conducted using Bio-Rad software.

### NO Measurement

Human umbilical vein endothelial cells were seeded in six-well plates at the appropriate densities. The cells were treated when they reached 70–80% confluence. HUVECs were then washed with phosphate-buffered saline (PBS) and incubated with 10 μM diaminofluorescein-FM diacetate (DAF-FM DA; NO-sensitive fluorescent dye) without phenol red at 37°C in 5% CO_2_ for 30 min. Measurement of NO production was performed using an Olympus microscope (IX71, Japan). The mean fluorescence intensity values were analyzed using ImageJ software.

The content of NO in mesenteric arteries was determined using the Griess method according to the instruction of the manufacturer. Briefly, tissues were lysed on ice and then centrifuged at 14,000 *g* at 4°C for 5 min. The supernatants were collected, and protein concentrations were determined by BCA method. The standard NaNO_2_ and samples were added to a 96-well plate (50 μl/well), respectively. After adding Griess Reagents I and II sequentially (50 μl of each reagent/well), the absorbance was determined at 540 nm in wavelength.

### PP2A Activity Assay

PP2A activity was measured using a V2460 kit from Promega (Madison, WI, United States) as previously reported (Sun et al., 2012). One 6-cm-dish of cells and 0.1 grams of mesenteric arterial tissue were lysed with 300 μl of precooled phosphatase storage buffer, lysed on ice for 30 min, and then centrifuged at 4°C and 12,000 rpm for 25 min to remove the supernatant. As per the manufacturer’s instructions, 250 μl of supernatant was added to the column. The filtrate was collected as the sample to be tested, and the phosphate content of the sample was measured after the reaction using the optical density value at 600 nm. The enzyme activity of PP2A in each sample was calculated based on a standard curve.

### Measurement of Intracellular ROS

Intracellular ROS production was measured using the ROS-sensitive detection probe 2,7-dichlorodihydrofluorescein diacetate (DCFH-DA). HUVECs were cultured in six-well plates and treated with the appropriate drugs when they reached 60–70% confluence. The following day, the cells were cocultured with 5 μM DCFH-DA in the dark at 37°C in 5% CO_2_ for 30 min. The cells were then washed three times with PBS and observed under an Olympus microscope. The mean fluorescence intensity values were analyzed using ImageJ software.

### p22phox Small Interfering RNA Transfection

HUVECs were seeded in six-well plates and cultured until they reached 40–50% confluence. Prior to transfection, culture medium containing 10% FBS was replaced with serum-free culture medium without antibiotics, and the cells were serum-starved for 2 h. A transfection reagent (Santa Cruz, CA, United States) was used to perform transfection of small interfering RNA (siRNA; Santa Cruz, CA, United States) p22phox gene into HUVECs, which was performed in six-well plates. The final concentration of p22phox or scrambled siRNA was 80 pmol per well. After transfection for 6 h, the medium was replaced with fresh serum-free culture medium without antibiotics.

### Statistical Analysis

Data were analyzed using SPSS version 17.0 statistical software, and were expressed as mean ± SD. Homogeneity of variance test was used to compare samples from multiple groups. A one-way ANOVA or two-way ANOVA was used for comparisons among groups. Values of *p* < 0.05 were considered to indicate statistically significance.

## Results

### AngII Induced eNOS Ser1177 Dephosphorylation Resulting in a Reduction in NO Production *via* AT_1_R

**Figure 1:**
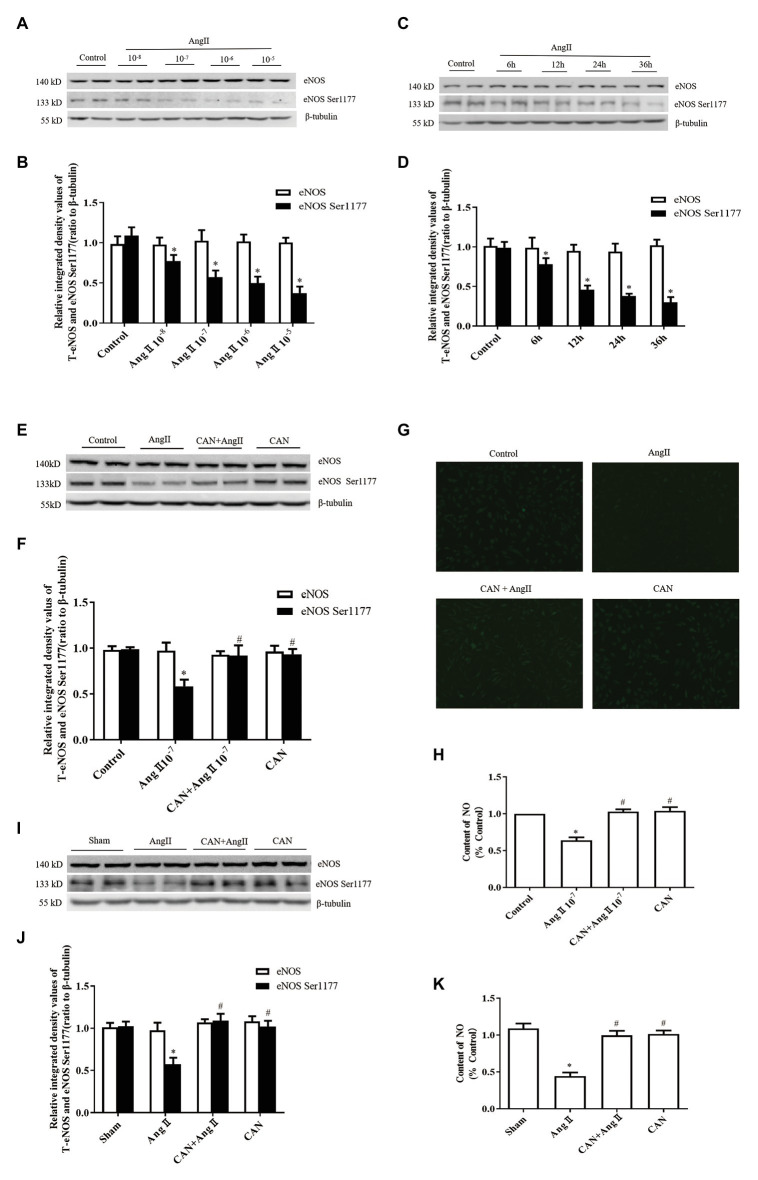
Effects of the angiotensin II (AngII)/angiotensin type 1 receptor (AT_1_R) pathway on endothelial nitric oxide synthase (eNOS) Ser1177 phosphorylation and nitric oxide (NO) production. **(A,B)** AngII downregulated phosphorylation levels of eNOS Ser1177 in a dose-dependent manner. Human umbilical vein endothelial cells (HUVECs) were treated with the indicated concentrations of AngII for 12 h, and phosphorylation levels of eNOS Ser1177 were detected by Western Blot analysis (*n* = 6 independent experiments). **(C,D)** AngII downregulated phosphorylation levels of eNOS Ser1177 in a time-dependent manner. HUVECs were treated with 10^−7^ M AngII for the indicated time, and phosphorylation levels of eNOS Ser1177 were detected by Western Blot analysis (*n* = 6 independent experiments). **(E,F)** Candesartan (CAN) remarkably inhibited AngII-mediated downregulation of eNOS Ser1177 in HUVECs. HUVECs were pretreated with CAN (10^−6^ M, 3 h) and then stimulated with AngII (10^−7^ M, 12 h), and phosphorylation levels of eNOS Ser1177 were detected by Western Blot analysis (*n* = 6 independent experiments). **(G,H)** CAN blocked AngII-mediated (10^−7^ M, 12 h) downregulation of NO production in HUVECs. HUVECs were incubated with DAF-FM DA (10 μmol/L) for 30 min. The representative images were captured with a fluorescence microscope (200× magnification; *n* = 4 independent experiments). **(I,J)** AngII reduced levels of eNOS Ser1177 phosphorylation in rat mesenteric arteries, whereas the AT_1_R antagonist, CAN, blocked AngII-mediated downregulation of eNOS Ser1177 phosphorylation (*n* = 6 rats per group). Rats received AngII infusion *via* osmotic minipumps for 2 weeks. CAN was administered after minipump implantation by gavage at a dose of 10 mg/kg/day during the AngII infusion period. **(K)** The content of NO in rat mesenteric arteries of AngII-infused rats. The content of NO was decreased in the mesenteric arteries of AngII-infused rats. CAN blocked AngII-induced downregulation of NO production (*n* = 3 rats per group). ^*^ *p* < 0.05 vs. Control or Sham group; ^#^ *p* < 0.05 vs. AngII group.

We investigated the effects of AngII/AT_1_R on phosphorylation of eNOS Ser1177 and production of NO in cultured HUVECs and AngII-infusion rats. In cultured HUVECs, after incubation with AngII at concentrations of 10^−5^, 10^−6^, 10^−7^, and 10^−8^ M for 12 h or at a concentration of 10^−7^ M for 6, 12, and 24, and 36 h, the phosphorylation levels of eNOS Ser1177 were significantly lower than those in the Control group (Figures 1A–D). Based on these results, treatment with 10^−7^ M AngII for 12 h was used for subsequent experiments. CAN, AT_1_R antagonist, abolished AngII-induced decrease of eNOS Ser1177 phosphorylation (Figures 1E,F). There were no statistically significant differences in total eNOS protein expression levels among the groups. To clarify the effect of AngII on NO generation, we used DAF-FM DA fluorescent probe to measure the NO content in HUVECs. The results showed that the NO production of the AngII group was lower than that of the Control group, and blocking AT_1_R with CAN reversed the production of NO (Figures 1G,H).

In AngII-infusion rats, the systolic blood pressure increased significantly after 3 days, reached the highest value at day 7, and remained at stable level until 14 days. Treatment the rats with CAN markedly reduced the systolic blood pressure in AngII-infusion group (Table 1). Next, we measured protein expression levels of eNOS and eNOS Ser1177 in the mesenteric arteries of rats at day 14. AngII infusion decreased the levels of phosphorylation of eNOS Ser1177, and CAN abolished the effect of AngII. There were no statistically significant differences in eNOS protein expression levels among the groups (Figures 1I,J). AngII infusion reduced the NO production, which could be reversed by CAN (Figure 1K). The alterations of the levels of eNOS protein expression, eNOS Ser1177 phosphorylation and NO content were consistent with those of in HUVECs. These results demonstrated that AngII dephosphorylates eNOS Ser1177 leading to a reduction in NO production *via* AT_1_R pathway.

**Table 1:** Systolic blood pressure in each group of rats.

Group	Before pump implanted (mmHg)	Day 3 after pump implanted (mmHg)	Day 7 after pump implanted (mmHg)	Day 14 after pump implanted (mmHg)
Sham (*n* = 12)	89.39 ± 3.09	86.80 ± 2.14	88.39 ± 3.03	87.27 ± 2.54
AngII (*n* = 12)	84.77 ± 2.81	125.12 ± 3.59^*^^#^	150.66 ± 5.05^*^^#^^△^	153.86 ± 2.21^*^^#^^△^
CAN + AngII (*n* = 12)	87.21 ± 2.62	89.69 ± 4.22^▲^	91.61 ± 4.33^▲^	95.50 ± 3.39^▲^
CAN (*n* = 12)	88.69 ± 1.66	86.05 ± 2.08	87.46 ± 4.17	89.00 ± 4.86

Effects of AngII infusion on the systolic blood pressure in each group rats. Data are presented as mean ± SD.

* *p* < 0.05 vs. Sham group.

# *p* < 0.05 vs. before pump implanted.

△ *p* < 0.05 vs. 3 days after pump implanted.

▲ *p* < 0.05 vs. AngII group.

### AngII/AT_1_R Pathway Downregulates the Phosphorylation of eNOS Ser1177 and Reduces the Production of NO by Activating PP2A

**Figure 2:**
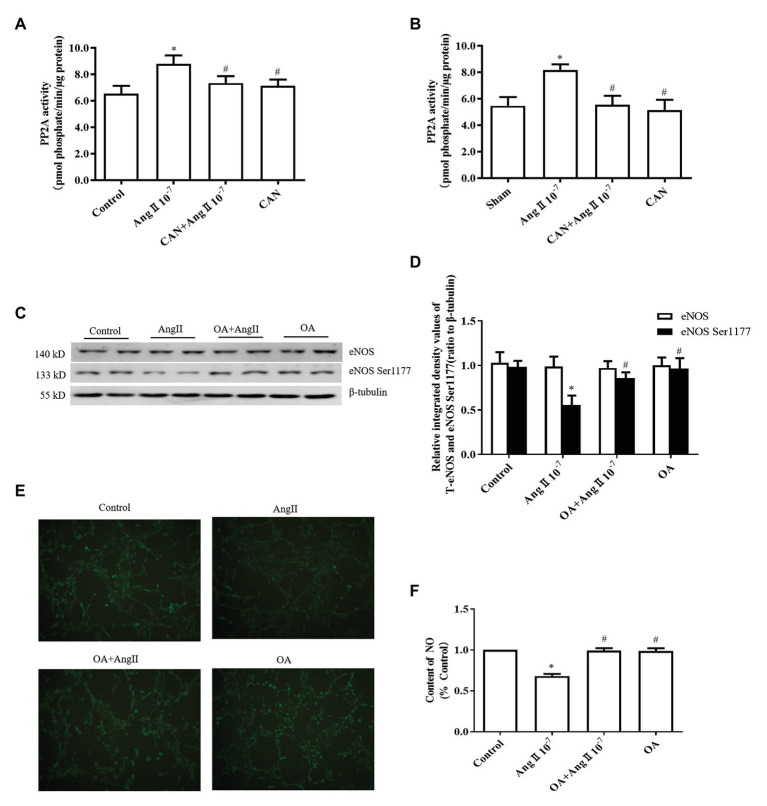
The AngII/AT_1_R pathway reduces the levels of eNOS Ser1177 phosphorylation and NO production by activating protein phosphatase 2A (PP2A). **(A)** AngII augmented PP2A activity, while CAN blocked AngII-mediated enhancement of PP2A activity. HUVECs were pretreated with 10^−6^ M CAN for 3 h or not, then stimulated with 10^−7^ M AngII for 12 h (*n* = 4 independent experiments). **(B)** PP2A activity was increased in the mesenteric arteries of AngII-infused rats. CAN blocked AngII-induced upregulation of PP2A activity (*n* = 3 rats per group). **(C,D)** PP2A inhibitor (OA) blocked the effect of AngII on eNOS Ser1177 *in vitro*. HUVECs were pretreated with 10^−8^ M OA for 1 h or not, then stimulated with 10^−7^ M AngII for 12 h (*n* = 6 independent experiments). **(E,F)** OA blocked AngII-induced downregulation of NO production in HUVECs (200× magnification; *n* = 4 independent experiments). ^*^p < 0.05 vs. Control or Sham group; ^#^*p* < 0.05 vs. AngII group.

It was reported that PP2A can dephosphorylate eNOS Ser1177 and decrease eNOS activity (Mount et al., 2007; Zhang et al., 2012). Therefore, we examined the activity of PP2A both *in vitro* and *in vivo*. As expected, the activity of PP2A was significantly increased both in the AngII-treated HUVECs and mesenteric arteries of AngII-infusion rats, pretreatment with CAN reduced the activity of PP2A (Figures 2A,B). To further clarify the role of PP2A in AngII-induced eNOS/NO dysfunction, 10^−8^ M PP1/PP2A inhibitor OA were used to pretreat HUVECs, according to the report that OA at concentration of 10^−8^ M inhibits PP2A enzyme activity (Chao et al., 2014). The results demonstrated that pretreatment with OA reversed the effect of AngII on phosphorylation of eNOS Ser1177 and generation of NO (Figures 2C–F). These findings indicated that AngII/AT_1_R activated PP2A resulting in dephosphorylation of eNOS Ser1177, decreased eNOS enzyme activity, and reduced NO production.

### Activation of the AngII/AT_1_R Pathway Upregulates PP2Ac Tyr307 Phosphorylation to Activate PP2A by Promoting ROS Generation

**Figure 3:**
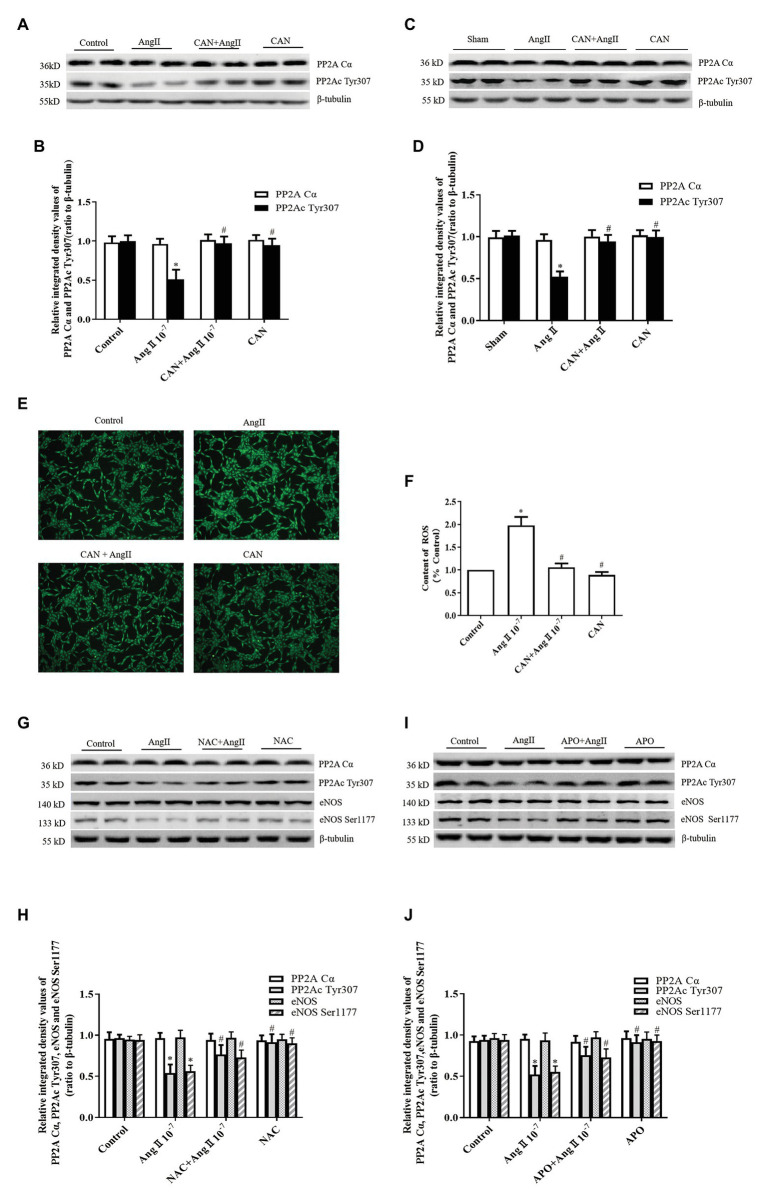
AngII/AT_1_R pathway downregulates PP2Ac Tyr307 phosphorylation, which is related with ROS generation. **(A,B)** CAN blocked AngII-mediated downregulation of PP2Ac Tyr307 phosphorylation in HUVECs. HUVECs were pretreated with 10^−6^ M CAN for 3 h or not, then stimulated with 10^−7^ M AngII for 12 h (*n* = 6 independent experiments). **(C,D)** CAN blocked AngII-mediated downregulation of PP2Ac Tyr307 phosphorylation in rat mesenteric arteries (*n* = 6 rats per group). **(E,F)** ROS content was enhanced by AngII (10^−7^ M, 12 h) and abolished by pretreatment with CAN (10^−6^ M, 3 h). HUVECs were incubated with DCFH-DA (5 μmol/L) for 30 min, and ROS content was measure. The representative images shown were captured using a fluorescence microscope (100× magnification; *n* = 4 independent experiments). Pretreatment with the antioxidant, **(G,H)** N-acetylcysteine (NAC; 10^−3^ M, 1 h) and **(I,J)** Apocynin (APO; 2 × 10^−5^ M, 1 h), inhibited AngII/AT_1_R-mediated downregulation of PP2Ac Tyr307 and eNOS Ser1177 (*n* = 6 independent experiments). ^*^p < 0.05 vs. Control or Sham group; ^#^*p* < 0.05 vs. AngII group.

Post-translational phosphorylation modulation of PP2A catalytic subunit, for instance, phosphorylation of PP2Ac Tyr307 reduces its activity (Ishii et al., 2017). Therefore, we measured the phosphorylation level of PP2Ac Tyr307. The results showed that AngII treatment significantly decreased phosphorylation of PP2Ac Tyr307, which could be prevented by pretreatment with CAN both *in vitro* and *in vivo* (Figures 3A–D). There were no statistically significant differences in the PP2A catalytic subunit α protein (PP2ACα) expression among the groups. Accordingly, AngII may activate PP2A by reducing the phosphorylation level of PP2Ac Tyr307.

AngII is one of the most common oxidative stress-induced factors. Therefore, we speculated that AngII/AT_1_R activated PP2A may be related to the increased production of ROS. We used the DCFH-DA fluorescent probe to measure the content of intracellular ROS. The results showed that the ROS production was higher in the AngII group than that in the Control group, and CAN treatment decreased the production of ROS (Figures 3E,F). To clarify the effect of ROS on AngII/AT_1_R-induced PP2A activation, we pretreated HUVECs with the antioxidants NAC and APO. The data showed that NAC and APO restored the levels of PP2Ac Tyr307 and eNOS Ser1177 phosphorylation (Figures 3G–J). Taken together, these data suggest that activation of the AngII/AT_1_R pathway promotes the production of ROS, which activates PP2A by downregulating the phosphorylation of PP2Ac Tyr307, leading to eNOS Ser1177 dephosphorylation.

### Effects of Nox on AngII/AT_1_R-Induced PP2A Activation

**Figure 4:**
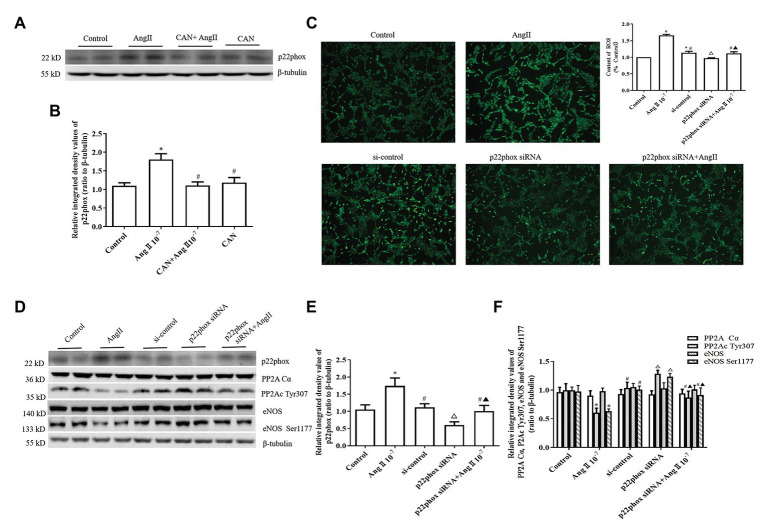
AngII downregulating the levels of PP2Ac Tyr307 phosphorylation is dependent on NADPH oxidase (Nox) activation and ROS production in HUVECs. **(A,B)** AngII (10^−7^ M, 12 h) augments p22phox protein expression and pretreatment with CAN (10^−6^ M, for 3 h) blocked the effect of AngII (*n* = 6 independent experiments). **(C)** Silencing of p22phox protein expression by siRNA abrogated AngII-mediated increase of ROS (100× magnification; *n* = 4 independent experiments). **(D–F)** Knockdown of p22phox protein by siRNA reversed the downregulation of PP2Ac Tyr307 and eNOS Ser1177 (*n* = 6 independent experiments). HUVECs were transfected with control siRNA or p22phox-specific siRNA for 24 h, then treated with 10^−7^ M AngII for 12 h. Levels of PP2Ac Tyr307 and eNOS Ser1177 were determined by Western blot analysis. ^*^p < 0.05 vs. Control group; ^#^*p* < 0.05 vs. AngII group; ^△^*p* < 0.05 vs. si-control group; ^▲^*p* < 0.05 vs. p22phox siRNA group.

NADPH oxidase is the main source of ROS in endothelial cells exposed to AngII (Cat et al., 2013). There have been reports suggest that the p22phox subunit is critical for the activation of Noxs except Nox5 and duox1/2 (Petry et al., 2010), and AngII increased the expression of p22phox and induced oxidative stress in the lungs and hearts of mice with hypoxia-induced pulmonary hypertension (Zhang et al., 2019). Therefore, we measured the protein expression level of p22phox in HUVECs. The results showed that AngII treatment augmented p22phox protein expression and CAN pretreatment blocked this effect of AngII (Figures 4A,B).

To further confirm the roles of Nox, HUVECs were transfected with p22phox siRNA. After p22phox gene silencing by p22phox siRNA, the amount of ROS decreased significantly (Figure 4C). Western blot analysis revealed that knockdown of p22phox protein expression by p22phox siRNA obviously upregulated the phosphorylation levels of PP2Ac Tyr307 and eNOS Ser1177. Moreover, p22phox knockdown almost abolished the effects of AngII on PP2Ac Tyr307 and eNOS Ser1177 phosphorylation in the HUVECs (Figures 4D–F).

## Discussion

Endothelial dysfunction is considered the basis of CVD, and eNOS/NO dysfunction is a common feature of ED (Godo and Shimokawa, 2017). NO is considered central to mediating the diverse action carried out by the endothelium and plays a pivotal role in regulating endothelium-dependent dilatation. Under physiological conditions, eNOS is the predominant source of NO in endothelial cells (Bonetti et al., 2003; Zhao et al., 2015). AngII is a key component of the renin–angiotensin system and regulates physiological and pathological cardiovascular functions mainly *via* its specific receptors (Tassone et al., 2013; Ding et al., 2016). Several studies have demonstrated that AngII contributes to the pathogenesis of ED by decreasing eNOS activity and NO bioavailability. It is reported that treatment of human aortic endothelial cells with AngII downregulates the phosphorylation of eNOS Ser1177, decreases eNOS activity and NO production by downregulating the expression of PGC-1α (Li et al., 2016). The previous study shows that AngII decreases eNOS activity and total NO content by upregulating SIRT3 expression in HUVECs (Liu et al., 2015). eNOS enzyme activity is regulated by various mechanisms, including protein post-translation phosphorylation. The eNOS phosphorylation site at serine 1177 near the carboxyl terminal is the center of eNOS activity regulation, phosphorylation at this site significantly upregulates eNOS activity, and PP2A is the major phosphatase that dephosphorylates eNOS leading to a decrease in eNOS activity and NO production (Searles, 2006; Fleming, 2009). However, the precise molecular mechanisms under which AngII activates PP2A to drop the levels of eNOS Ser1177 phosphoryation are remained unknown. In the present study, we demonstrated that AngII downregulates eNOS Ser1177 phosphorylation by activating PP2A *via* the AT_1_R/Nox/ROS signaling pathway.

PP2A is a highly conserved serine/threonine phosphatase that exists across species as a dephosphorylation protein (Janssens and Goris, 2001). It is composed of structural subunit A, regulatory subunit B, and catalytic subunit C. PP2A regulates several important cellular processes, such as cell cycle, apoptosis, cell metabolism, and migration, *via* dephosphorylation of intracellular proteins (Apostolidis et al., 2016; Wlodarchak and Xing, 2016). Studies have shown that PP2A is activated in CVD, and that activated PP2A could lead to ED *via* dephosphorylation of eNOS or Akt (Zhang et al., 2012; Etwebi et al., 2018; Schnelle et al., 2019). AngII/AT_1_R can enhance the activity of PP2A in cardiomyocytes (Everett et al., 2001) as well as HUVECs (Luo et al., 2019). In the present study, we observed that AngII/AT_1_R pathway activation increased PP2A enzyme activity and reduced phosphorylation of eNOS Ser1177 and the content of NO both *in vitro* and *in vivo*; PP2A inhibition (by OA) reversed the phosphorylation of eNOS Ser1177 *in vitro*. These results suggested that AngII/AT_1_R downregulates phosphorylation of eNOS Ser1177 by activating PP2A.

There are several mechanisms involved in the modulation of PP2A activity, including enzyme assembly, subunit post-translational modification, inhibitors, and protein interactions. The PP2A catalytic subunit, PP2Ac, can be modified by phosphorylation, methylation, and acetylation (Lambrecht et al., 2013; Hung et al., 2016). We previously reported that AngII/AT_1_R activates PP2A by reducing endogenous phosphatase 2A inhibitor 2 (I_2_^PP2A^; Luo et al., 2019). Phosphorylation of the PP2Ac at Tyr307 site inactivates PP2A; therefore, phosphorylation level of PP2Ac Tyr307 is considered to be the indicators of PP2A activity (Brautigan, 1995; Ishii et al., 2017). In the present study, we found that the phosphorylation level of PP2Ac Tyr307 decreased and PP2A activity increased after AngII treatment accompanied by the decline in the levels of eNOS Ser1177 and NO generation. All of these changes were reversed by OA *in vitro*. These results suggest that downregulation of PP2Ac Tyr307 phosphorylation is one of mechanisms by which the AngII/AT_1_R pathway increases PP2A activity. However, the molecular mechanisms involved in AngII-induced PP2A activation were needed to further explore.

Oxidative stress is an established cause of ED, and has been well recognized in the pathogenesis of CVD. The NADPH oxidase system is one of the main sources of ROS. There are seven known members of the Nox family: Nox1, Nox2, Nox3, Nox4, Nox5, Duox1, and Duox2. The main isoforms of Nox in endothelial cells are Nox1, Nox2, Nox4, and Nox5 and are the major sources of endothelial cell-derived ROS (Touyz et al., 2011; Wingler et al., 2011; Cat et al., 2013). It has been confirmed that AngII activates Nox and promotes the production of ROS (Qiu et al., 2015; Liu et al., 2016; Zhang et al., 2019). It is believed to increase ROS production *via* the Nox families by increasing their protein expression as well as their catalytic activity (Zhang et al., 2019).

ROS leads to endothelial cell damage through multiple pathways, including activation of proinflammatory signaling pathways, depletion of antioxidants and signaling molecules, and oxidation of macromolecules (Valko et al., 2007; Incalza et al., 2018). In addition, ROS results in the uncoupling of eNOS, which leads to the reduction of NO production and bioavailability in endothelial cells (Drummond and Sobey, 2014) and cardiomyocytes (Roe et al., 2013). NO is the key molecule to regulate the biological function of endothelium. The reduced NO bioavailability is a significant mechanism of ROS induced endothelial damage. eNOS is the main source of endothelial NO. eNOS oxidizes the terminal guanidine nitrogen atom of L-arginine by using electrons from NADPH to produce NO when the substrate (L-arginine) and co-substrate (tetrahydrobiopterin, flavin adenine mononucleotide, flavin adenine dinucleotide, and NADPH) are sufficient. The eNOS protein is a homodimer, and dimerization is necessary for NO production. Uncoupled eNOS is unable to deliver electrons between two monomers, then electrons from NADPH can be captured by oxygen and produced superoxide anion (O_2_^−^; Bonetti et al., 2003; Roe and Ren, 2012; Zhao et al., 2015). Whether ROS activates a protein phosphatase to reduce the eNOS enzyme activity through post-translation phosphorylation modulation is an important purpose of this study.

Previous studies have shown that PP2A is a target molecule of Nox-derived ROS (Nagata et al., 2006; Menden et al., 2013). Therefore, we hypothesized that activation of PP2A by AngII/AT_1_R may be related to Nox/ROS activation. Our study confirmed that AngII/AT_1_R notably increased ROS formation and p22phox protein expression in HUVECs, and the effect of AngII on PP2Ac Tyr307 and eNOS Ser1177 phosphorylation was eliminated after ROS reducing by NAC and APO. Nox enzyme complex comprises of membrane bound subunits (p91phox and p22phox) and cytoplasmic subunits (p47phox, p67phox, p40phox, and Rac). Phosphorylated cytoplasmic subunits form a complex and translocate to the membrane to dock with the membrane subunits (Panday et al., 2015). p22phox is one of the two membrane subunits, and regulates the activity of Nox. Therefore, we further verified the effect of Nox/ROS on PP2A activation by knocking down p22phox gene expression *in vitro*. After p22phox gene silencing, the ROS production reduced significantly, and the AngII-induced dephosphorylation of PP2Ac Tyr307 and eNOS Ser1177 decreased. Accordingly, we believe that AngII/AT_1_R activates PP2A by downregulating the phosphorylation of PP2Ac Tyr307 leading to the losses of eNOS Ser1177 phosphorylation and NO production at least in part through the Nox/ROS signaling pathway. However, the mechanism by which Nox/ROS causes the downregulation of PP2Ac Tyr307 is not well understood, and it may be related to the tyrosine protein kinases (Fedida-Metula et al., 2012; Xiong et al., 2013), which need to be confirmed in the next work.

**Figure 5:**
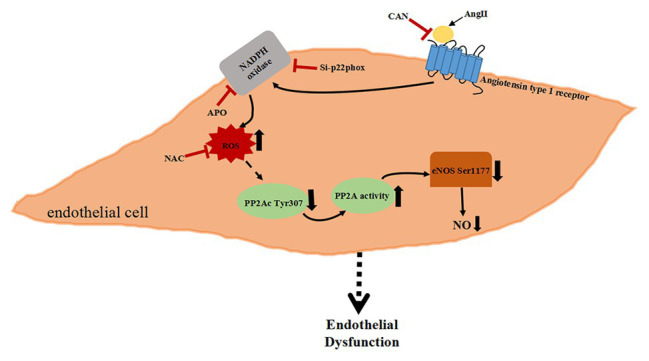
Schematic diagram of AngII-activated PP2A downregulate eNOS phosphorylation *via* the Nox/ROS signaling pathway.

In summary, the present study demonstrates that AngII binding to its specific type 1 receptor activates PP2A through Nox/ROS signal pathway, which leads to eNOS/NO dysfunction. The increased Nox membrane subunit p22phox protein expression causing excessive ROS activates a certain signaling pathway to decrease the phosphorylation of PP2Ac Tyr307 further increase the activity of PP2A. PP2A dephosphorylated eNOS Ser1177 and reduced NO production, which may be another significant mechanism of AngII induced ED (Figure 5).

## Data Availability Statement

All data included in this study are available upon by contact with the corresponding authors.

## Ethics Statement

The studies involving human participants were reviewed and approved by The Ethics Committee of Guizhou Medical University. Written informed consent to participate in this study was provided by the participants’ legal guardian/next of kin. The animal study was reviewed and approved by Guizhou Medical University Animal Care and Use Committee.

## Author Contributions

JD has performed all experiments and revised the manuscript. MY has performed all experiments, analyzed all data, and drafted the manuscript. JJ performed the animal experiments. YL helped with *in vitro* experiments and data analysis. SW and FY helped with collection of the data. QZ helped with the Western blotting analysis. AW and LW helped with the NO measurement. MZ helped with the data analysis and revised the manuscript. QZ made the statistical charts. SW and YX revised the manuscript. DL designed the study, supervised all experiments, and responsible for critically revising the manuscript. All authors contributed to the article and approved the submitted version.

### Conflict of Interest

The authors declare that the research was conducted in the absence of any commercial or financial relationships that could be construed as a potential conflict of interest.

## Abbreviations

AngII: Angiotensin II
PP2A: Protein phosphatase 2A
AT_1_R: Angiotensin type 1 receptor
Nox: NADPH oxidase
ROS: Reactive oxygen species
HUVECs: Human umbilical vein endothelial cells
CAN: Candesartan
NAC: N-acetylcysteine
APO: Apocynin
OA: Okadaic acid
siRNA: Small interfering RNA
ED: Endothelial dysfunction
NO: Nitric oxide
eNOS: Endothelial nitric oxide synthase
CVD: Cardiovascular disease
